# Risk factors and management of different types of biliary injuries in blunt abdominal trauma: Single-center retrospective cohort study

**DOI:** 10.1016/j.amsu.2020.02.009

**Published:** 2020-03-10

**Authors:** Hazem M. Zakaria, Ahmed Oteem, Nahla K. Gaballa, Osama Hegazy, Ali Nada, Talaat Zakareya, Hazem Omar, Hazem Abdelkawy, Hesham Abdeldayem, Emad Hamdy Gad

**Affiliations:** a Department of Hepatopancreatobiliary and Liver Transplant Surgery, National Liver Institute, Menoufia University, Menoufia, Egypt; b Department of Anesthesiology and Intensive Care, National Liver Institute, Menoufia University, Menoufia, Egypt; c Department of Hepatology and Gastroenterology, National Liver Institute, Menoufia University, Menoufia, Egypt; d Department of Diagnostic and Intervention Radiology, National Liver Institute, Menoufia University, Menoufia, Egypt

**Keywords:** Blunt liver trauma, Biliary injury, Bile leak, ERCP

## Abstract

**Background:**

Biliary injuries after blunt abdominal traumas are uncommon and difficult to be predicted for early management. The aim of this study is to analyze the risk factors and management of biliary injuries with blunt abdominal trauma.

**Method:**

Patients with blunt liver trauma in the period between 2009 to May 2019 were included in the study. Patients were divided into 2 groups for comparison; a group of liver parenchymal injury and group with traumatic biliary injuries (TBI).

**Results:**

One hundred and eight patients had blunt liver trauma (46 patients with liver parenchymal injury and 62 patients with TBI). TBI were; 55 patients with bile leak, 3 patients with haemobilia, and 4 patients with late obstructive jaundice. Eight patients with major bile leak and 12 patients with minor bile leak had been resolved with a surgical drain or percutaneous pigtail drainage. Nineteen patients (34.5%) with major and minor bile leak underwent successful endoscopic retrograde cholangiopancreatography (ERCP). Sixteen patients (29.1%) underwent surgical repair for bile leak. In Multivariate analysis, the possible risk factors for prediction of biliary injuries were central liver injuries (*P* = 0.032), high grades liver trauma (*P* = 0.046), elevated serum level of bilirubin at time of admission (*P* = 0.019), and elevated gamma glutamyl transferase (GGT) at time of admission *(P* = 0.017).

**Conclusion:**

High-grade liver trauma, central parenchymal laceration and elevated serum level of bilirubin and GGT are possible risk factors for the prediction of TBI. Bile leak after blunt trauma can be treated conservatively, while ERCP is indicated after failure of external drainage**.**

## Highlights

• In most of the published series they discussed the iatrogenic biliary injuries or injuries after sharp trauma.
• To our knowledge it is the largest series to discuss the biliary injuries with blunt liver trauma.
• We can predict the possible risk factors for bile duct injury after blunt liver trauma.
• So we can diagnose and treat it properly and early before sepsis and biliary complications.
• We can approach to the ideal treatment modality for each type of biliary injuries with prober timing.

## 1 Introduction

The liver is the most common solid organ to be injured after blunt abdominal trauma. About 80–90% of biliary injuries occur after sharp penetrating traumas like stab wounds or gunshots. But biliary injuries after blunt traumas are uncommon; it was reported in 2.8–7.4% of patients**.** It is predominantly caused by road traffic accidents (RTA), kicks or falls [1,2].

The site of biliary tract injuries may involve; intrahepatic bile ducts that are associated with parenchymal lacerations, extrahepatic bile ducts as occurs at points of anatomic fixation, and gall bladder (GB) injury especially with distended GB. There is a challenge in the diagnosis of biliary injuries and needs a high index of suspicion as the usual images are not specific. So, the diagnosis usually comes during exploratory surgery or after the development of late complications [[3], [4], [5]].

The management of traumatic biliary injury (TBI) is comparatively more challenging than the management of iatrogenic biliary injuries (IBI) due to associated organ injuries and the delayed diagnosis. Most of the patients with blunt liver trauma have minor bile leaks with a good response to conservative treatment. However, the management of major bile leak is a difficult dilemma and can affect patient recovery. The time of diagnosis and the effective method of intervention is the key in the management of major bile leaks. Early treatment with the diversion of the bile flow can help in the prevention of the development of infected biloma and intraabdominal sepsis. Percutaneous pigtail drainage and successful endoscopic retrograde cholangio-pancreatography (ERCP) with stenting are the cornerstones in the treatment of bile leaks with blunt abdominal trauma [[6], [7], [8]].

Most of the studies discussed the IBI or biliary injuries associated with penetrating trauma, but few reports in the literature discussed this subject in detail, with a limited number of patients with biliary injuries after blunt traumas. The study aimed to analyze the management of different types of biliary injuries after blunt abdominal traumas, the safety and efficacy of non-operative management, and possible factors for early detection of biliary injuries with blunt trauma.

### 1.1 Patients and methods

We conducted a retrospective cohort study to patients with blunt abdominal traumas who were admitted to our department of hepato-pacreato-biliary (HPB) surgery, National Liver Institute (NLI), Menoufia University, Egypt, in the period between 2009 and May 2019. Data were collected from the prospectively collected database and approved by the local institutional review board (IRB) (NLI: 23,745). All the patients assigned informed consent before surgery to use their related prospective database as needed for research work. The research was registered according to declaration of Helsinki in the Chinese clinical trial registry with registration number ChiCTR2000029020.

Our inclusion criteria were all blunt liver trauma patients who subsequently underwent damage control surgery or conservative treatment and developed biliary complications like; biliary leak, haemobilia, and/or biliary stricture. Patients with liver injuries were resuscitated and initially treated in their primary trauma centers according to the principles of advanced trauma life support Hemodynamic instability or signs of peritonitis were the main indications for an exploratory laparotomy. (Our hospital is a tertiary referral center for HPB surgery so, all patients were initially treated in a trauma center and some of them received maneuvers before their referral). Liver injuries were detected in computed tomography (CT) scan, or during abdominal exploration.

The patients with blunt abdominal trauma were divided into two groups for comparison; a group with only liver parenchymal injury after blunt abdominal trauma, and the other group with associated biliary injuries. The following data were collected; demographic data, mechanism of blunt trauma (motor vehicle or bike accident, fall from height, Fights or kicks and heavy compression). The Interval between the onset of trauma and admission to our hospital, any previous maneuver was done before referral to our hospital e.g. exploratory laparotomy, laparoscopic drainage.

American Association for the Surgery of Trauma (AAST) classification system was used for grading of liver injury [9], high-grade trauma was considered ≥ grade III. Injury Severity Score (ISS) was used to assess the severity of the trauma. Major trauma or polytrauma is defined as the ISS being greater than 15 [10].

All patients with biliary injury after blunt abdominal trauma were identified and included in this study. Post TBI subdivided into bile leakage (biloma or biliary fistula), hemobilia or bile duct stricture according to the images or clinical presentation.

Liver injuries were further divided into central (involving segments 4, 5, or 8), peripheral (involving segments 2, 3, 6, or 7), or based on abdominal CT. Traumatic bile duct injuries were classified into intrahepatic and extrahepatic. Intrahepatic biliary duct injuries were divided into central (falls within 5 cm from the hepatic duct bifurcation), or peripheral (falls within the hepatic parenchyma >5 cm from the confluence of hepatic ducts) [5,6].

A biliary fistula was defined as leakage of bile via the surgical wound, or percutaneous drain of more than 50 mL/day, confirmed by a bilirubin fluid level more than the normal bilirubin level in the serum. According to (Hommes et al., 2014), bile leaks were classified as minor or major leaks. A major leak was defined as drainage of greater than 400 mL/d or persistent drainage of greater than 50 mL/d for longer than 14 days. A minor leak was defined as drainage of bile of less than 400 mL/d or greater than 50 mL/d for not longer than 14 days [11].

Surgical repair was classified to early repair in the first 2 weeks after the trauma, and late repair after 6 weeks.

The follow up of the patients was from the day of the admission or surgery to December 2019, the median period of follow up was 56 months.

## 2 Statistical analysis

Statistical analysis was done using the SPSS software (v.20, IBM, New York, USA). The two groups of patients were compared using Fisher's exact or Chi-square tests for categorical variables, and for continuous non-normally distributed variables we used Mann-Whitney *U* test. A Logistic regression test was used for multivariate analysis to compare between the two groups. P values less than 0.05 were considered statistically significant.

## 3 Results

**Fig. 1:**
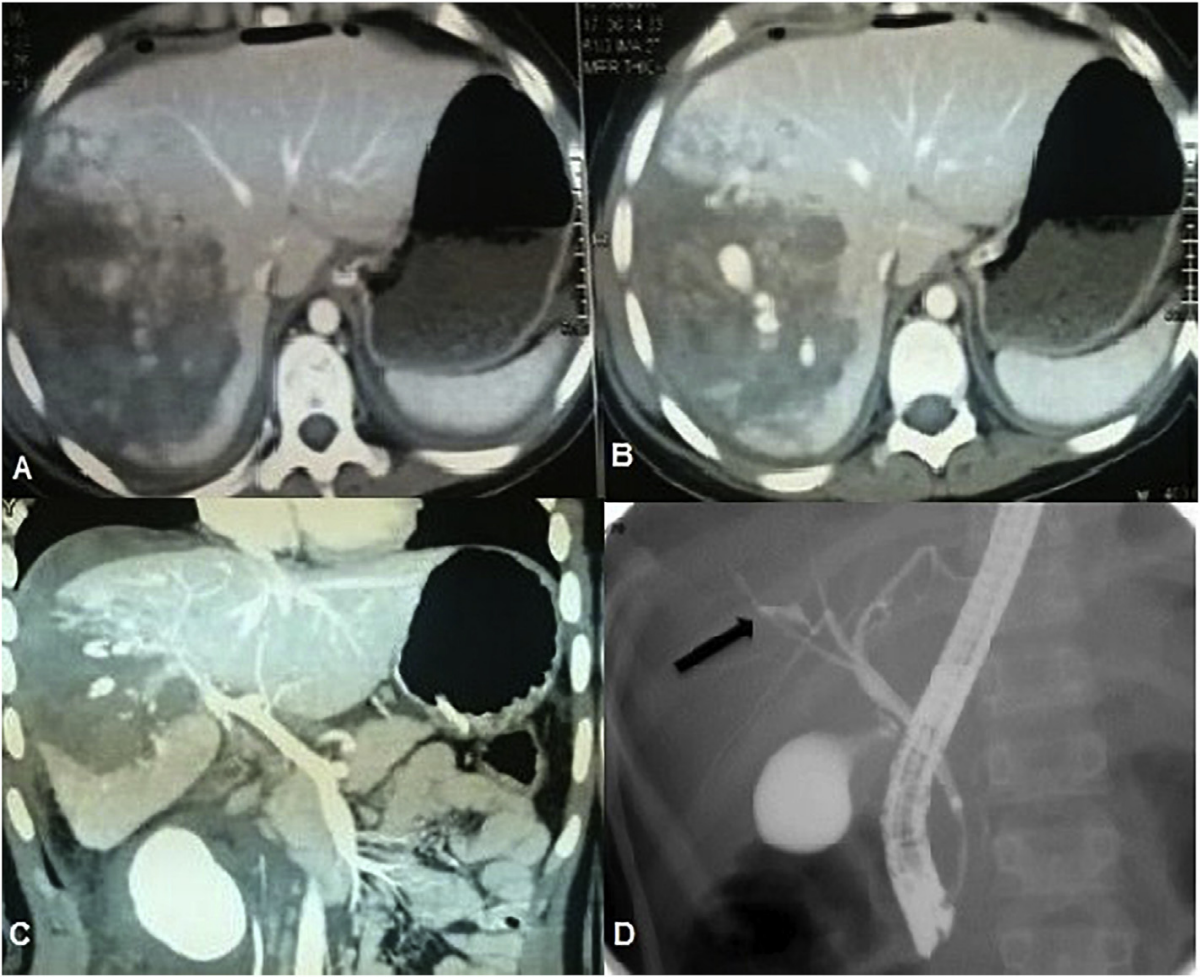
A,B,C) CT pictures of female patient 12 years old with RTA blunt abdominal trauma grade V, she had lacerated right hepatic lobe with large hematoma involving segments 6,7,8, intraparenchymal extravasation of the dye is seen. D) ERCP demonstrating bile leak (arrow indicates bile leak), of Rt anterior duct.

**Fig. 2:**
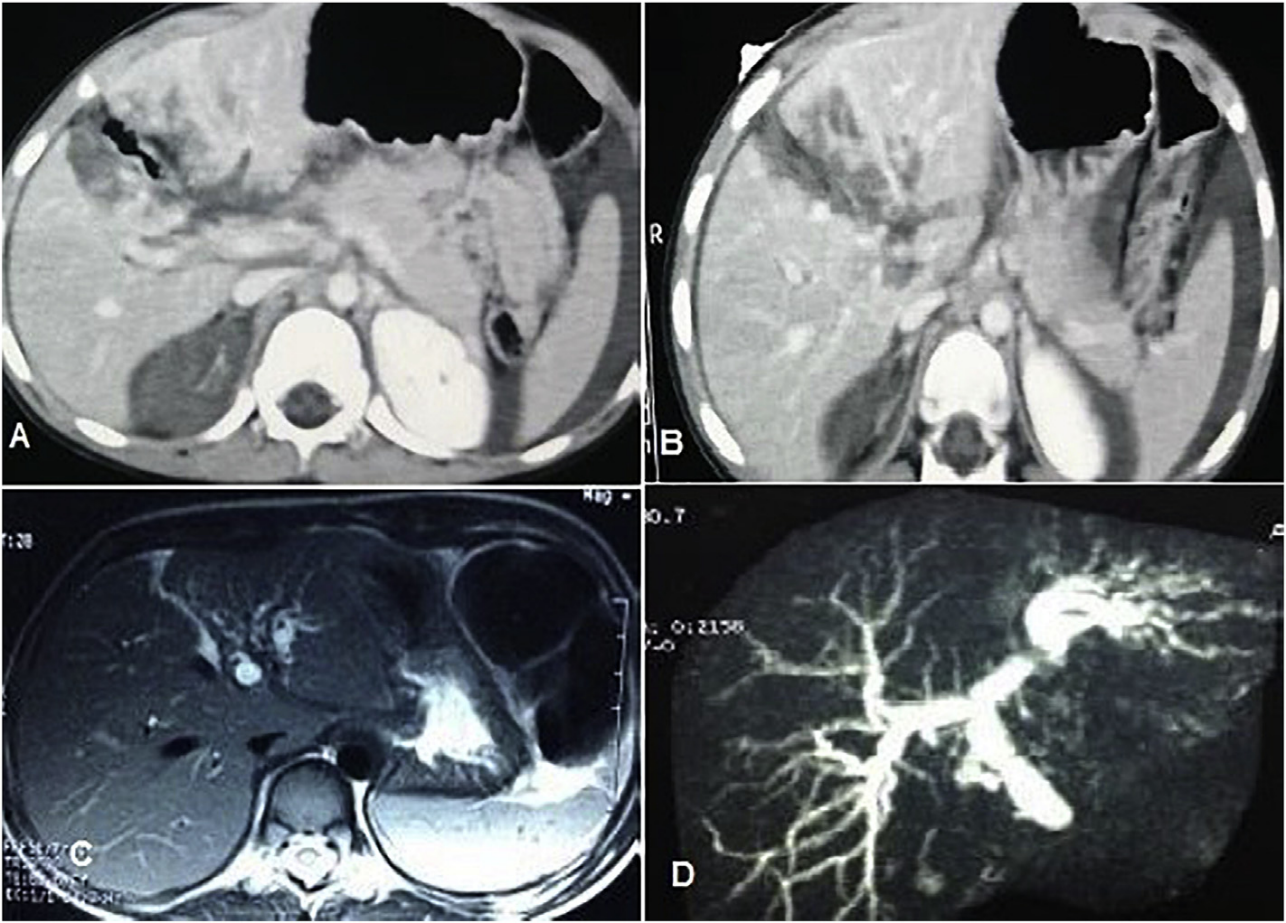
A, B) CT shows grade IV trauma, large complex tear about 9 cm involving the central portion of the liver segment III, IV, VIII and it branches laterally to involve segment V, VI and medially to involve caudate lobe. C, D) MRCP after 5 months of the trauma showed strictures at left hepatic duct and CHD with subsequent dilated biliary radicles.

**Fig. 3:**
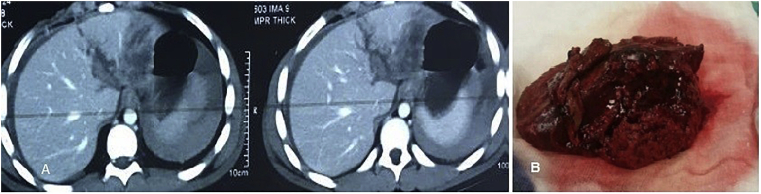
A) CT images of male patient, 12 years old, grade IV trauma to the left lateral sector of the left lobe, exploration after failure of conservative treatment revealed major bleeding from portal vein in the umbilical fissure and left lateral resection was done, then patient developed minor bile leak from the drain that resolved spontaneously. B) Operative image of the resected left lateral part.

**Fig. 4:**
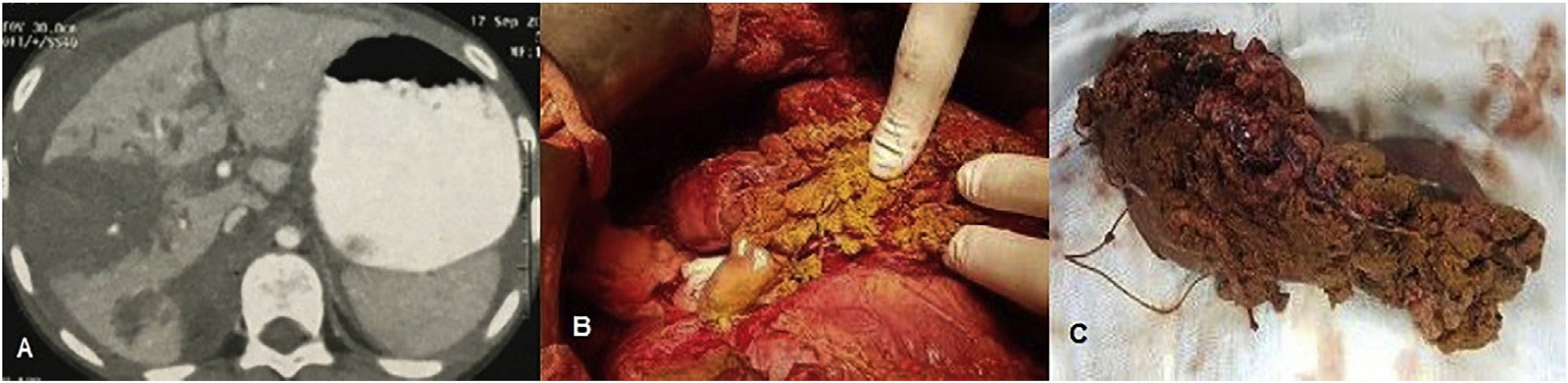
A) CT image of male patient with RTA, explored before admission and perihepatic packing was done for lacerated Rt lobe, referred to us and was explored for control of ongoing bleeding and removal of packing. B) Surgical debridement with closure of suspicious small bile ducts on cut surface with proline 5/0 and drain inserted. C) The resected part of Rt lobe.

**Fig. 5:**
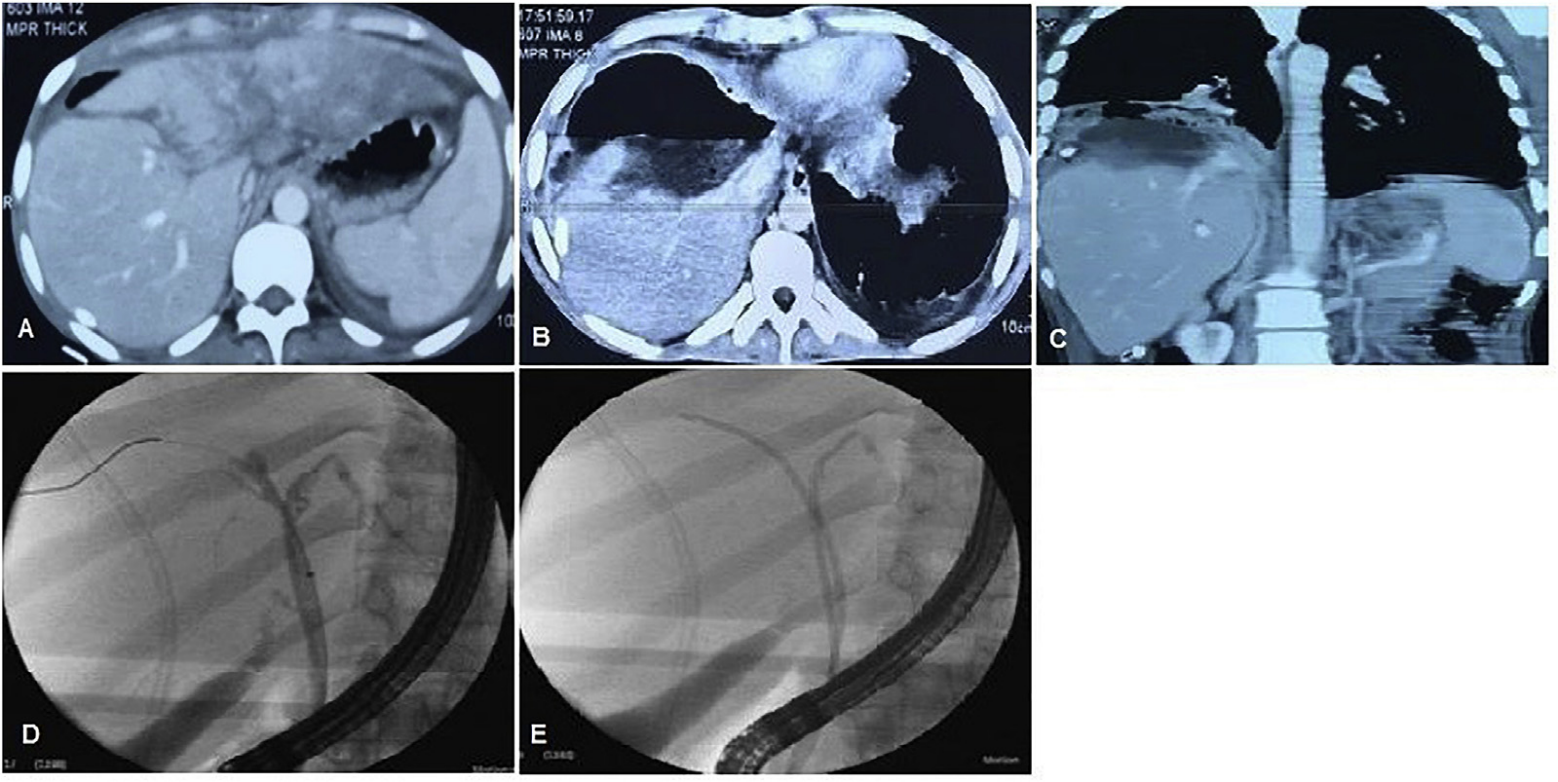
A) CT image of male patient, 20 years old, RTA blunt trauma, shows grade IV liver trauma, B,C) large fluid collection at site of Segments 4 & 8 with air fluid level due to liver abscess complicating central trauma with subsequent broncho-biliary fistula (BBF) & left lobe congestion. D) ERCP images for management of left BBF. E) Both the right and left biliary were cannulated and two 7F 15 cm plastic stents were applied and the patient improved with un eventful follow up course.

One hundred and eight patients who had blunt abdominal trauma were included in our study, 62 patients (57.4%) had associated TBI, and 46 patients (42.6%) had only liver parenchymal injury. RTA with right upper abdominal crush trauma was the main cause of abdominal trauma (Table 1). Most of the patients with associated biliary injuries were exposed to major trauma in 79% of patients and mean ISS 17 (Fig. 1, Fig. 2, Fig. 3, Fig. 4, Fig. 5). The site of liver injury was mainly central in 48.4% of patients with biliary injury and the right lobe in 60% (Fig. 1, Fig. 2, Fig. 4).

**Table 1:** Preoperative, operative and postoperative data for both groups of blunt abdominal trauma.

	Group1 associated TBI (No = 62)	Group2 liver parenchymaL injury (No = 46)	P value
Age (year)			0.87
mean ± SD	17 ± 12	16 ± 10	
range	(4-45)	(6-39)	
Adults	32(51.6%)	21(45.7%)	0.76
Pediatrics	30(48.4%)	25(54.3%)	
Sex			0.8
Male	44(71%)	32(69.6%)	
Female	18(29%)	14(30.4%)	
Mechanism of blunt trauma			0.43
RTA trauma or Run over accident	38(61.3%)	33(71.7%)	
Fall from height	9(14.5%)	6(13%)	
Hit by hard object	5(8.1%)	4(8.7%)	
Others	10(16.1%)	3(6.5%)	
Site of liver injury			**0.02**
Central	30(48.4%)	6(13%)	
Peripheral	13(21%)	35(76.1%)	
Central and peripheral	14(22.6%)	5(10.9%)	
No liver parenchymal injury	5(8%)	0	
AAST			0.07
No parenchymal injury	5(8%)	0	
Grade II	8(12.9%)	29(63%)	
Grade III	30(48.3%)	13(28.3%)	
Grade IV	16(25.8%)	4(8.7%)	
Grade V	3(4.8%)	0	
High grades liver trauma	49(79%)	17(37%)	**0.0**1
Type of biliary injury			
Intrahepatic	44 (71%)	0	
Extrahepatic	11(17.7%)	0	
Intra- and extrahepatic	7(11.3%)	0	
Injury severity score	17 ± 9	15 ± 8	0.08
<15	24(38.7%)	26(56.5%)	
Serious>15	29(46.8%)	15(32.6%)	
Severe>25	9(14.5%)	5(10.9%)	
Interval between trauma and referral (days)	12 ± 16	4 ± 5	**0.01**
Early(within 7 days)	30(48.4%)	43(93.5%)	
Late(>7days)	32(51.6%)	3(6.5%)	
Sepsis			0.08
No	38(61.3%)	41(89.1%)	
Yes	24(38.7%)	5(10.9%)	
Blood transfusion			0.09
No	26(41.9%)	31(67.4%)	
Yes	36(58.1%)	15(32.6%)	
Total Bilirubin (mg/dl) at our admission			**0.001**
mean ± SD	4.2 ± 3.6	1 ± 0.9	
Normal	12(19.4%)	40 (87%)	
Elevated	50(80.6%)	6(13%)	
ALT (IU/L)			0.07
mean ± SD	53 ± 64	62 ± 46	
Normal	26(41.9%)	4(8.7%)	
Elevated	36(58.1%)	42(91.3%)	
GGT(IU/L)			**0.001**
mean ± SD	97 ± 54	41 ± 38	
Normal	13(21%)	41(89.1%)	
Elevated	49(79%)	5(10.9%)	
Conservative management ± pigtail drainage			**0.015**
No	39(77.6%)	8(17.4%)	
Yes	23(22.4%)	38(82.6%)	
TAE			1.0
No	58(93.5%)	43(94.7%)	
Yes	4(6.5%)	3(5.3%)	
ERCP			0.001
No	33(51.6%)	46(100%)	
Yes	31(48.4%)	0(0%)	
Stent			0.04
No	37(59.7%)	46(100%)	
Yes	25(40.3%)	0(0%)	
Surgical exploration after trauma			0.42
No	41(66.1%)	38(82.6%)	
Yes	21(33.9%)	8(17.4%)	
Surgical intervention to biliary system			
No	44(71%)		
Yes	18(29%)		
-for major bile leak	16	0	
-for late H-J to obstructive jaundice (at 6 &12 months post trauma).	2	0	
ICU stay			0.08
mean ± SD	4 ± 10	2 ± 3	
range	(2-15)	(1–7)	
Hospital stay			**0.001**
mean ± SD	25 ± 9	9 ± 5	
range	(6-95)	(4-28)	
Early postoperative complications			0.12
No	38(73.1%)	43(93.5%)	
Yes	14(26.9%)	3(6.5%)	
Early readmission			0.07
No	42(67.7%)	43(93.5%)	
Yes	20(32.3%)	3(6.5%)	

Statistical tests used: Mann-Whitney *U* test, Fisher's exact test.

TBA (traumatic biliary injury), RTA (road traffic accident), AAST (American Association for the Surgery of Trauma, SD (standard deviation), ALT (alanine transaminase), GGT (gamma glutamyl transferase), TAE (trans arterial embolization), ERCP (endoscopic retrograde cholangiopancreatography), H-J (hepatico-jejunostomy) ICU (intensive care unit).

Our hospital is a tertiary center for HPB surgery so, all patients were initially treated in a trauma center before referral to our hospital. Surgical laparotomy was done in 29 patients (26.9%) in the primary trauma center. The main initial operative management was perihepatic packing (n = 3), hepatorrhaphy, hemostasis by diathermy, argon or foam gel (n = 13), laparoscopic exploration and abdominal drainage (n = 4). Associated intra-abdominal injuries included kidney (n = 3), spleen (n = 4), pancreas (n = 2).

After our hospital admission; seven patients with major bleeding underwent control by trans-arterial embolization (TAE) of branches of right or left hepatic arteries.

**Fig. 6:**
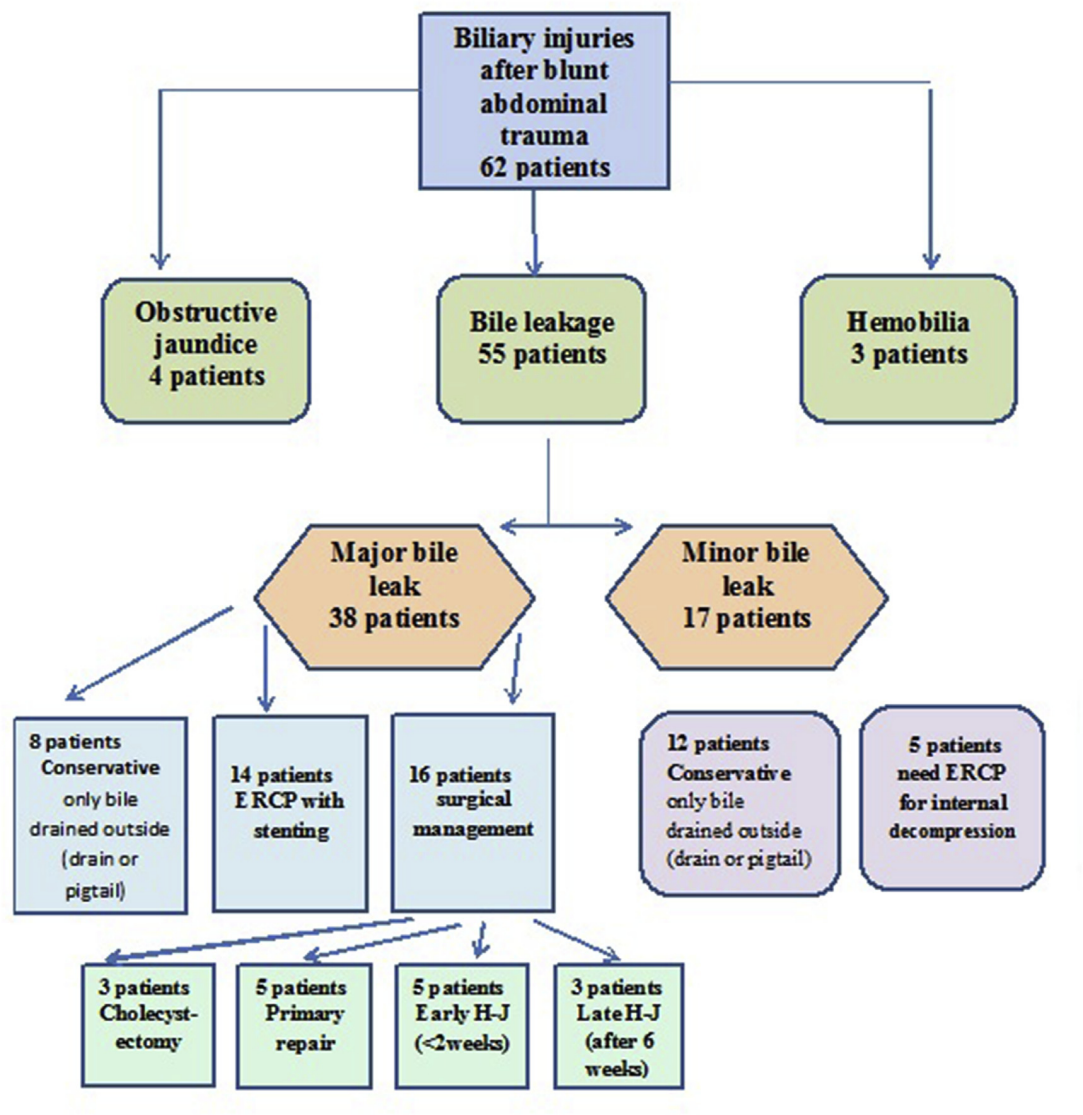
Diagram of patients with traumatic biliary injuries and their management.

The incidence of TBI among the study population was 62 patients 57.4%; 55 patients presented with biliary leakage, with mean time of diagnosis of bile leak was 5 days post-trauma (range: 2–30 days), three patients presented with hemobilia and four patients presented late with obstructive jaundice (OJ) (Fig. 6).

Seven patients (11.3%) of TBI had isolated bile duct injury after blunt abdominal trauma without liver parenchymal injury.

Two patients with haemobilia had a history of grade III&IV right lobe laceration with exploration and hepatorrhaphy. After 3–4 weeks of trauma, they complained of haematemesis, melena, and OJ. CT revealed post-traumatic pseudoaneurysm with branches of the right hepatic artery. Management was by TAE, with uneventful follow up course. One patient had mild haemobilia and self-controlled within few days after trauma.

Four patients had a history of grade II or III liver trauma and after 6–12 months presented with OJ as the first presentation; one patient resolved after 3cessions of percutaneous transhepatic cholangiography (PTC) and dilatation of common bile duct (CBD) stricture. One patient had a choledocho-gastric fistula and distal CBD stricture that was diagnosed by PTC and it was resolved after 4 sessions of ERCP and stenting. Two patients underwent Roux-en-Y hepatico-jejunostomy (H-J) repair after 6 and 12 months of trauma due to common hepatic duct (CHD) tight stricture and choledocho-duodenal fistula, respectively.

Fifty-five (50.1%) patients with post-traumatic bile leak; 38 patients had major bile leak and 17 patients had a minor bile leaks. Management was as in (Fig. 6). Sixteen patients (25.8%) had a surgical intervention to the biliary leak; three patients had ruptured GB and underwent cholecystectomy, five patients underwent liver resection of the traumatic liver segments (Fig. 3, Fig. 4) and primary repair of CHD with trans*-*cystic duct stent, five patients underwent early H-J and three patients underwent late H-J.

Two patients after surgical repair hade bile leak which stopped spontaneously on conservative treatment. Six patients had recurrent cholangitis and biliary stricture later on after control of biliary leak with meantime 7 ± 6 months after the primary procedure [1 patient after early surgical repair, 1 patient after late surgical repair (*P* = 0.6), and 4 patients after previous control of bile leak by ERCP and stenting (*P* = 0.23)]. These patients underwent management by ERCP with repeated stenting (n = 2) and H-J (n = 4).

Two patients after exploration for liver trauma and bile leak complicated by right lobe abscess and subphrenic abscess then broncho-biliary fistula (BBF), they underwent pigtail drainage of the abscess and multiple ERCP with stenting then fistula closed after about 2–4 months (Fig. 5).

There is no peri-operative mortality case in our study population. 1-, 3-, and 5- year survival rates were all 100%.

In univariate analysis, there was significant difference between the both groups (Table 1) in site of liver injury (*P* = 0.02), high grades liver trauma (*P* = 0.01), time of referral to our hospital (*P* = 0.01), elevated serum level of bilirubin at time of admission (*P* = 0.001), and elevated Gamma-glutamyl transferase (GGT) enzyme at time of admission (*P* = 0.001), conservative management (*P* = 0.015), hospital stay (*P* = 0.001). In Multivariate analysis, the possible risk factors from which we can be able to predict presence of biliary injuries and manage them early were central liver injuries (OR = 1.37, 95% CI: 1.16–1.62, *P* = 0.03), high grades liver trauma (OR = 1.97, 95% CI: 2.13–4.82, *P* = 0.046), elevated serum level of bilirubin at time of admission (OR = 2.33, 95% CI:0.59–9.24*, P* = 0.019), and elevated GGT at time of admission (OR = 2.7, 95% CI: 1.2–5.7, *P* = 0.017).

## 4 Discussion

In the recent decades with the introduction of “damage control” strategy and non-operative management (NOM) for haemodynamically stable patients with blunt liver trauma, the mortality rates have sharply decreased, but in expense of increased liver-related complications like biliary tree disruption with collection of biloma or persistent bile leak [1,11,12].

Although there is now substantial experience in the management of IBI, the management of TBI is still a difficult dilemma as it is difficult to treat the patients with the same algorithm used for IBI. The basic principles of repair have to be tailored for every patient according to the clinical presentation after trauma [11]. To our knowledge, it is the largest series in biliary injuries with blunt abdominal trauma.

According to Tinkoff et al., RTA was the most common cause of liver injury in blunt abdominal trauma [13]*,* as we reported in our study. Yaman et al., reported that conservative treatment was the most successful management in many patients with blunt hepatic injuries whatever the degree of injury or the volume of hemoperitoneum [14], and these findings go parallel to that of our study as NOM was done in 79 patients (73.1%) after blunt hepatic trauma. Interventional arteriography with selective embolization of the bleeding vessel, radiological guided percutaneous drainage of infected collections, endoscopic sphincterotomy or biliary stenting are successful in the management of most of the complications in up to 85% of patients with blunt trauma [11].

The site of biliary tract injury after blunt abdominal trauma can be intrahepatic or extrahepatic, while the extrahepatic type can occur in the absence of any fracture injury to the liver parenchyma [2,4,6], and these findings match with our study as 11.3% patients with a biliary injury did not have any parenchymal fracture.

Kulaylat et al., reported that a high index of suspicion should be present in patients with selective NOM who develop symptoms such as nausea, abdominal distention, and pain after an initial period of clinical improvement after the trauma [12,15]. The delay time between the diagnosis of biliary tract injury and the onset of trauma varies greatly between days to months. For instance, Eid et al., reported a case with partial left hepatic duct tear that was diagnosed on day 9 after the hospital admission and Miyayama et al., confirmed a case with bile duct disruption and biloma after 46 days of the initial presentation [16,17]. In our analysis, the mean duration between the onset of trauma and the diagnosis of bile leak among the study population was 5 days ± 6.2, range (2–30 days). Keil et al., showed that delayed bile leaks could be discovered after the rupture of a sub-capsular collection or ischemia of the bile duct, so early laparotomy does not exclude the subsequent bile leakage [18]. Yuan et al., and Wahl et al., reported that high-grade liver trauma ≥ grade IV, central parenchymal injuries, non-selective TAE for bleeding and elevated serum level of bilirubin, were the risk factors for major bile leak after blunt abdominal trauma [6,19], and in our study, high-grade liver injury, centrally located parenchymal injuries, elevated serum bilirubin level and GGT were the main risk factors for TBI. According to Yuan et al., peripherally located liver injuries are less liable to develop major bile leak than the centrally located main bile ducts with better recovery than the later ones [6]. Pandey et al., showed that extrahepatic biliary injuries occur commonly at the relatively more fixed areas like; the bifurcation of the hepatic ducts, the origin of the left duct, and at the junction with the pancreatic duct [3], these findings go parallel to that of our study.

Hommes et al., had a series of 51 patients with bile leak after abdominal trauma, they reported 26 patients with minor bile leaks and 14 patients (35%) with a major leaks. They concluded that conservative management of bile leaks is safe and most of the bile leak can resolve spontaneously within 14 days, provided that it is adequately drained by the surgical drain or the percutaneous drainage. They considered also that ERCP is not a benign procedure, and internal biliary drainage should only be considered if bile drainage is more than 400 mL/day or if the leak is persistent after 14 days [11]. Lubezky et al., in their study also concluded that ERCP together with percutaneous drainage represent an effective and safe strategy in the treatment of bile leaks following hepatic trauma [20]. These findings go parallel to that of our study as 20 patients (36.4%) had successful drainage of bile leak by the surgical drains or by percutaneous pigtail drainage and 19 patients (34.5%) had successful ERCP for control of bile leak.

The key to the treatment of major bile leak is timely diagnosis and effective intervention. Early treatment with bile flow diversion can prevent the development of further complications such as biloma infection or even intra-abdominal sepsis [6,20] as we described in our results.

Omar and Redwan reported that surgical treatment was reserved for the management of 39 patients (25.2%) with complex bile leak, and early referral for the specialized centers in hepatobiliary surgery can save the lives of many patients and limit the morbidity and mortality [21]. Hommes et al., also reported that surgical repair was needed in only 11patients (22%) with post-traumatic bile leak [11]. In our study, 16 patients (29.1%) underwent surgical repair for bile leak.

Some series showed that early surgical repair of biliary injuries had equal outcomes to late repair [22,23], in our study 10 patients with early surgical repair of the biliary leak had a good postoperative outcomes.

TBI may have biliary strictures as a late presentation after months to years of the primary trauma. It is most probably related to the failure of the previous attempts of repair, or long term ischemia to biliary ducts. Currently, Roux-en-Y HJ is the most popular and successful surgical repair [4,24]. In the present study, there were 4 patients with the first presentation of biliary strictures and OJ after 6–12 months of trauma, it may be due to traumatic ischemia to bile ducts, and 6 patients with late biliary stricture after previous attempts of ERCP or surgical management.

Srivastava et al., reported that hemobilia can appear as a late complication with the presentation of abdominal pain, haematemesis, melena or OJ. The condition may be minor and can stop spontaneously, or sever as in cases with associated post-traumatic pseudoaneurysm that needs percutaneous arteriography by selective microembolization of the arterial branches that communicate with the biliary system [25]. In our series had two patients who presented late with hemobilia and treated with selective TAE of the right hepatic artery pseudoaneurysm with uneventful follow-up course.

According to Eryigit et al., broncho-biliary fistula can be presented by cough and bilioptysis. Several mechanisms are suggested for the development of BBF, including sub-diaphragmatic abscess with subsequent ruptures into the bronchial system after the erosion of the diaphragm, leading to a communication between the bronchial tree and biliary channels. BBF is a serious complication after the blunt trauma with a high mortality and morbidity rates and requires a preliminary planned management strategy [26]. Chua et al., reported that whole treatment modalities for BBF fail if there is still obstruction in the main biliary tree and persistence of the fistula with a patent biliary channel is an indication for lung resection or thoracotomy [26,27]. These findings match with our analysis as we had two patients who suffered grade IV laceration of the central part of the liver parenchyma, then developed major bile leak that complicated with right lobe and subphrenic abscess. They were managed by endoscopic decompression and percutaneous pigtail drainage of sub-diaphragmatic and right lobe abscesses, bilious expectoration improved and stopped after 2–4 months.

Spontaneous formation of the biliary-enteric fistula is a rare sequel after TBI, it is often a consequence of severe trauma with associated duodenal or pancreatic injury [3]. In this study there were two patients with choledocho-duodenal fistula and choledocho-gastric fistula with distal CBD stricture, one patient underwent successful repair by H-J, and the other one resolved after multiple ERCP.

## 5 Conclusion

High-grade liver injury, central parenchymal laceration and elevated serum level of bilirubin and GGT enzyme at the time of admission are possible risk factors for the development of bile duct injury after blunt liver trauma. Most of the patients with bile leak after blunt trauma can be treated conservatively with radiological guidance percutaneous drainage, while ERCP with biliary stenting is indicated for those patients with expanding or persistent bilomas with failed resolution after conservative external drainage. Early surgical intervention is effective and Roux-en-Y- HJ is the standard surgical management for biliary leak or stricture after the failure of endoscopic treatment with good outcome in a specialized center with HPB surgery.

### 5.1 Provenance and peer review

Not commissioned, externally peer reviewed.

## Statement of ethics

The research was conducted ethically in accordance with the World Medical Association Declaration of Helsinki. The patients have given their written informed consent on admission and pre-operative to use their prospective data base and files for research work. The study protocol was approved by the National Liver Institute committee and review board (NLI: 23,745).

## Financial support

No**.**

## Consent

The research was conducted ethically in accordance with the World Medical Association Declaration of Helsinki. The patients have given their written informed consent on admission and pre-operative to use their prospective data base and files for research work (and as it is a retrospective study on the previous patients data and records so no need for new consents).

## Author contribution

(Hazem Zakaria, Ahmed Oteem, Nahla Gaballa, Osama Hegazy, Ali Nada, Talaat Zakareya, Hazem Omar, Hazem Abdelkawy, Hesham Abdeldayem) all actively participated in the preparation, study design, collection of the data and editing of the manuscript. Statistical analysis was done by Hazem Zakaria, and final revision approved by all authors.

## Registration of research studies

Name of the registry: Chinese Clinical Trial Registry.

Unique Identifying number or registration ID: ChiCTR2000029020.

Hyperlink to the registration (must be publicly accessible):

http://www.chictr.org.cn/edit.aspx?pid=47891&htm=4

http://www.chictr.org.cn/listbycreater.aspx.http://www.chictr.org.cn/listbycreater.aspx.

## Declaration of competing interest

No conflicts of interest.
